# Effective Water Use Required for Improving Crop Growth Rather Than Transpiration Efficiency

**DOI:** 10.3389/fpls.2018.01442

**Published:** 2018-09-28

**Authors:** Thomas R. Sinclair

**Affiliations:** Department of Crop and Soil Sciences, North Carolina State University, Raleigh, NC, United States

**Keywords:** crop growth, effective water use, stomatal conductance, transpiration, vapor pressure deficit

## Abstract

The phenomenological expression showing crop yield to be directly dependent on crop transpiration use efficiency (TE) has encouraged continued focus on TE as a viable approach to increasing crop yields. The difficulty in the phenomenological perspective is that research tends not to match up with the underlying mechanistic variables defining TE. Experimental evidence and the mechanistic derivation of TE by Tanner and Sinclair showed that the common focus on increasing the intrinsic ratio of leaf CO_2_/H_2_O exchange has limited opportunities for improvement. On the other hand, the derivation showed that daily vapor pressure deficit (VPD) weighted for the daily cycle of transpiration rate has a large, direct impact on TE. While VPD is often viewed as an environmental variable, daily weighted VPD can be under plant control as a result of partial stomatal closure during the midday. A critical feature of the partial stomatal closure is that transpiration rate is decreased resulting in conservation of soil water. The conserved soil water allows late-season, sustained physiological activity during subsequent periods of developing water deficits, which can be especially beneficial during reproductive development. The shift in the temporal dynamics of water use by water conservations traits has been shown in simulation studies to result in substantial yield increases. It is suggested from this analysis that *effective water use* through the growing season is more important for increasing crop yield than attempts focused on improving the static, intrinsic TE ratio.

## Introduction

There continues to be great interest in increasing crop transpiration efficiency (TE), which is often defined as crop mass production per unit of crop transpiration. This interest seems to be sustained in spite of the fact that more than a century of research has shown little progress in improving basic TE. This was pointed out by Tanner and Sinclair (1983) in their review of much of the research beginning early in the last century showed little evidence in progress toward increasing TE. The one noted exception has been the development from carbon isotope discrimination observations of the wheat cultivar ‘Drysdale’ in Australia for rainfed conditions (Rebetzke et al., 2002). However, the carbon isotope discrimination approach in itself did not resolve the exact physiological advantage of this variety. The percent yield increase of Drysdale was found to be less than 11% at a base yield of about 1 t ha^−1^ (i.e., yield improvement of 0.11 t ha^−1^) and the percent yield increase declined linearly with higher base yields.

**FIGURE 1:**
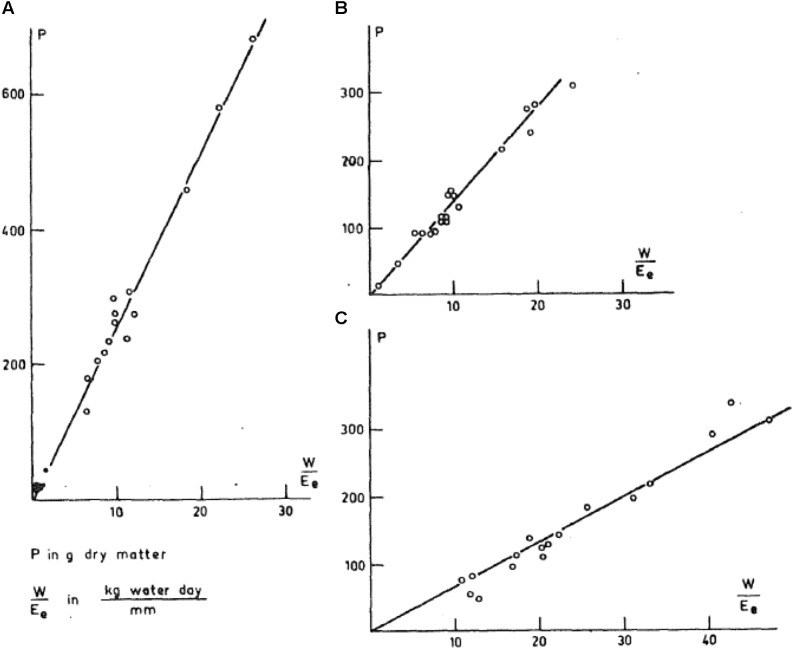
Graph of plant mass production plotted against transpiration rate normalized with pan evaporation (deWit, 1958). **(A)** Sorghum, **(B)** wheat, and **(C)** alfalfa.

Stability in TE was fully illustrated in the analysis of C.T. deWit (1958) in which results from experiments worldwide were combined and plotted for each species as growth vs. transpiration normalized by evaporation from an open water surface. These data within a species represented a range of cultivars, soil fertility, soil water conditions, and environments. As shown in **Figure 1**, within in each species these straightforward graphs resulted in highly linear relationships. The slopes varied among species but within species the slopes were extremely stable across the wide range of experimental conditions.

Given the historical experience of little variation within a species for improvement in the ratio of growth to normalized transpiration, why is there such a continuing interest in improving crop TE? Likely one major reason is the intuitive view that increasing TE will result in increased crop yield. This view was illustrated in the phenomenological equation presented by Passioura (1977).

(1) Y=HI×TE×W,

where Y = grain yield,

HI = harvest index,
W = transpired soil water.

A central feature of Eq. [1] is the TE variable, and this indicates that Y would be increased by increasing TE. The difficulty is that the Eq. [1] is not a *mechanistic* equation. As discussed below, TE is dependent on a large number of physical and physiological variables that make it very difficult to resolve TE in attempts at genetic comparisons and improvements.

## A Mechanistic View

Rather than the ambiguity in the phenomenological description of TE in Eq. [1], an improved understanding of canopy water use is obtained by examining a mechanistic description of TE. Such a mechanistic derivation was presented more than 30 years ago by Tanner and Sinclair (1983). In their derivation, the relationship between canopy mass accumulation and water loss was developed from the basic relation between carbon dioxide and water vapor exchange at the leaf level. The resultant expression resulted in the following deceptively simple expression for daily canopy TE.

(2) $$\text{TE}=\int (k_{\text{d}}/\text{VPD})\text{dt}/\int \text{dt},$$

where

*k*_d_ = mechanistic coefficient accounting for physical and physiological characteristics (Pa),
VPD = vapor pressure deficit (Pa).

If TE is to be calculated on a daily time step, then the daily value of VPD to be used in Eq. [2] must be weighted to reflect the daily pattern of transpiration rate. That is, the weighted VPD value needs to be skewed for the times of the day when transpiration rate is high. Therefore, a simple mean daily VPD even if based only on daytime values is an inappropriate calculation for daily VPD. Tanner and Sinclair (1983) proposed that VPD is equal to 0.75 of the difference between maximum daily vapor pressure and minimum daily vapor pressure. Abbate et al. (2004) subsequently concluded that the weighting coefficient in the calculation of VPD for Argentine environments was 0.72.

The terms that define the parameter *k*_d_ in Eq. [2] is critical to understanding the nature of TE. The derivation of Tanner and Sinclair (1983) gave the following explicit definition of *k*_d_.

(3) $$k_{\text{d}}=(\text{a}\times \text{b}\times \text{c}/1.5)\times ({\text{C}}_{\text{a}}/(\rho \times \varepsilon ))\times {\text{L}}_{\text{D}}/{\text{L}}_{\text{T}},$$

where

a = molecular weight ratio [CH_2_O]/[CO_2_] = 0.68,
b = conversion fraction to plant mass from hexose, *c* = (1-C_i_/C_a_), where C_i_ is leaf internal CO_2_ partial pressure and C_a_ is atmospheric CO_2_ partial pressure,
1.5 = accounting for diffusion difference between water vapor and CO_2_,
ρ = air density,
ε = ratio of mole weight of water vapor to air (18/28.8 = 0.625),
L_D_ = leaf area index exposed to direct radiation (for nearly closed canopies ∼1.4),
LT = effective transpiring leaf area index (for nearly closed canopies ∼2.2).

In comparing *k*_d_ among crop species, the two key variables that result in differences in *k*_d_ are parameters b and c. The value of b ranges from about 0.75 for species producing high carbohydrate plant products to about 0.42 for species producing high energy products containing high amounts of oil and protein. The value of c depends on the photosynthetic pathway of a species with maximum values of about 0.7 for C4 species and about 0.3 for C3 species. Therefore, *k*_d_ can range from about 9–10 Pa for carbohydrate-producing C4 species to about 4 Pa for energy-rich C3 species.

Based on the derived definition of *k*_d_, predicted *k*_d_ could be expected to be fairly stable within species. This conclusion is fully consistent with the stability in the slope in **Figure 1** from the analysis of deWit (1958). Further, the values of the slopes found by deWit reflect species differences in *k*_d_. That is, the species with the highest slope in **Figure 1** is sorghum, which is predicted to have a high slope because it is a C4 species producing low energy vegetative mass and seeds. The next lower slope is wheat, which is a C3 species producing high carbohydrate plant material. The lowest slope is for alfalfa, which is a C3 species producing high protein concentration in the plant.

The definition of *k*_d_ indicates some possibility of increasing TE by decreasing C_i_. There are two approaches to achieve decreased C_i_: lower stomatal conductance allowing C_i_ to be taken to a low value by photosynthetic consumption of CO_2_, or high leaf photosynthetic activity that results in the ready assimilation of CO_2_ and low C_i_. However, in the context of crop production neither approach seems likely to offer major opportunities for increases in TE. Low stomatal conductance will result in low leaf photosynthetic rates, which might result in a direct limitation on crop growth and yield. High leaf photosynthetic activity would likely be more advantageous, but for any crop species that has been subjected to breeding and selection for high yield, it seems likely that genotypes with low photosynthetic rates have already been discarded.

Direct measures of C_i_/C_a_ tend to show values that are consistent with the maximum *c* values given above. Bunce (2005) measured in the field the C_i_/C_a_ ratio of six C4 species and found the lowest ratio was in Japanese bristlegrass (*Seteria faberi*) with a ratio of 0.3 (maximum *c* = 0.7). In a comparison of a large number of sugarcane (*Saccharum* spp.) cultivars, Jackson et al. (2016) found the minimum C_i_/C_a_ to be 0.34 (maximum *c* = 0.66). In C3 species, the value of C_i_/C_a_ is much higher than in C4 species. In a field comparison of eight soybean [*Glycine max* (Merr.) culitvars, L.] at different stages of development, Tomeo and Rosenthal (2017) found the lowest value of C_i_/C_a_ to be about 0.66 (maximum *c* = 0.34) with most observations in the range about 0.69 (maximum *c* = 0.31) to 0.79 (maximum *c* = 0.21). In a comparison of seven cotton (*Gossypium hirsutum* L.) cultivars, C_i_/C_a_ ranged from 0.66 to 0.68 (maximum *c* = 0.34 to 0.32) (Stiller et al., 2005). Hence, the maximum experimental values of c in C4 and C3 are consistent with the stated values in the definition of *k*_d_ among species.

Given that both variables b and c are approximately stable with few practical options for major modification, it is concluded that the *k*_d_ term of TE does not appear to be a major priority target for increasing TE. The variable left for increasing TE is VPD. Unfortunately and importantly, VPD is often ignored in comparisons of TE even though it is clear that this variable can have a large impact on TE. Further, VPD is not simply defined by the changing ambient environmental conditions, but can be a physiological term resulting from plant adjustments in stomatal conductance over time and environmental conditions (Vadez et al., 2014).

Remembering that the VPD term in Eq. [2] represents daily VPD weighted for transpiration rate, the value of this term is decreased if the fraction of daily transpiration under high VPD conditions is decreased. There are two major approaches to result in decreased transpiration during the midday period of elevated VPD. One approach is the possibility of limited-transpiration rate due to the imposition of a maximum water transport to the guard cells due to plant hydraulic conductance limitations (Sinclair, 2017b). Under this condition, further increases in VPD result in partial stomatal closure so that the transpiration rate matches the water flow rate to the stomata. If there was not a limitation on transpiration rate due to partial stomatal closure, the leaf would rapidly desiccate due to limited water flow into the leaf. This stomatal response is sometimes observationally referred to as ‘midday stomatal closure.’ Sinclair et al. (2008) found that the limited-transpiration trait in soybean genotype PI 416937 was associated with low leaf hydraulic conductance, which was consistent with apparent aquaporin activity of this genotype (Sadok and Sinclair, 2010). Not surprising, in a study of peanut genotypes, Devi et al. (2009, 2010) found that those lines exhibiting partial stomata closure at threshold VPDs also had significantly greater TE.

A second approach to decreasing daily VPD can result from a decrease partial stomatal closure as the soil dries. Decreasing soil hydraulic conductance with soil drying results in partial stomatal closure at midday when no more than one third or less of the transpirable soil water remains in the soil (Sinclair, 2005). If a genotype with a low plant hydraulic conductance is paired with low soil conductance resulting from soil drying, then the threshold of extractable soil water at which transpiration decrease is likely initiated is at a higher soil water content than the usual one third transpirable soil water (Sinclair, 2017a). Those genotypes that initiate stomatal closure at high transpirable soil water will have a lower weighted VPD, and consequently greater TE as the soil dries.

## Decrease in Water Use by Environmentally Sensitive Stomatal Regulation

Given that there appears to be limited possibilities for increasing *k*_d_ in crop species that have been subjected to breeding for yield increase, a more rewarding focus for increasing TE seems likely to be on plant traits associated with decreased effective VPD (Eq. [2]). An increase in TE as a result of decreased weighted VPD certainly indicates a major opportunity for yield increase as shown in the Eq. [1]. However, Eq. [1] represents a static view of crop yield and fails to account for the temporally dynamic processes of mass accumulation and water use through an entire growing season. Accounting for the dynamic changes in water use through the growing season is critical in resolving the impact of VPD. Not only does weather directly influence VPD, but fluctuating availability of soil water can have a major influence on weighted VPD. Further, variation through the season on possible crop transpiration rate can influence the determination of weighted VPD.

In terms of increasing crop yield, an important outcome of the two water-conservation traits discussed above is that they result in altered seasonal patterns of water use. Conservation of soil water, especially early in the growing season, can result in greater soil water availability later in the growing season so that the impact of late-season drought might be decreased as a result of sustained physiological activity, especially during seed fill. Richards and Passioura (1989) selected wheat genotypes with smaller diameter metaxylem vessels as an approach to achieve decreased plant hydraulic conductance and shift water use to later in the growing season. While they found yield increases of 3 – 11%, no commercial cultivars were released from their study

A concern for each of the two water-conservation traits discussed here is that partial stomatal closure to limit water loss also results in a restriction on current photosynthetic activity. A key question to be resolved is whether the gain in conservation of soil water (and increased TE) overcomes the early season loss in plant mass accumulation. This question cannot be resolved using a static equation such as Eq. [1] but requires a temporal analysis through the growing season requiring a dynamic, mechanistic crop model. The model needs to be applied over a number of seasons for each location to obtain enough simulation results to allow adequate information to generate average yields, and likely more importantly, probability estimates for yield change.

Simulations to assess the yield response by introducing the water-conservations traits into crop genotypes have been done using the Simple Simulation Model (SSM, Soltani and Sinclair, 2012). This model tracks soil water content on a daily basis by adding precipitation and irrigation to the soil and removing water as a result of soil evaporation and canopy transpiration. The daily amount of crop mass accumulation, transpiration, leaf area development, and nitrogen accumulation are all adjusted in SSM based on the fraction of transpiration soil water (FTSW) that exists in the soil on each day as the simulation progresses through the growing season. Hence, the simulations are temporally dynamic and directly account for plant responses to soil water status.

### Assessment of Limited-Transpiration on Crop Yield

The impact of water conservation due to decreased transpiration rate under elevated VPD was first simulated for sorghum (*Sorghum bicolor* L.) in Australia (Sinclair et al., 2005). Weather data from four locations over more than 100 years was used to simulate sorghum plants with assumed, hourly limited-transpiration rates of 0.4 and 0.6 mm h^−1^. Simulated yields were generally increased, or at least unchanged, at yield levels of about 4.5 t ha^−1^ and lower. Approximately 75 % of the growing seasons were in this lower-yield classification that would benefit from the limited-transpiration trait. Above 4.5 t ha^−1^, yields were only slightly decreased due to the limited-transpiration trait. It was concluded that the limited-transpiration trait appeared advantageous for commercial production of sorghum in Australia.

**FIGURE 2:**
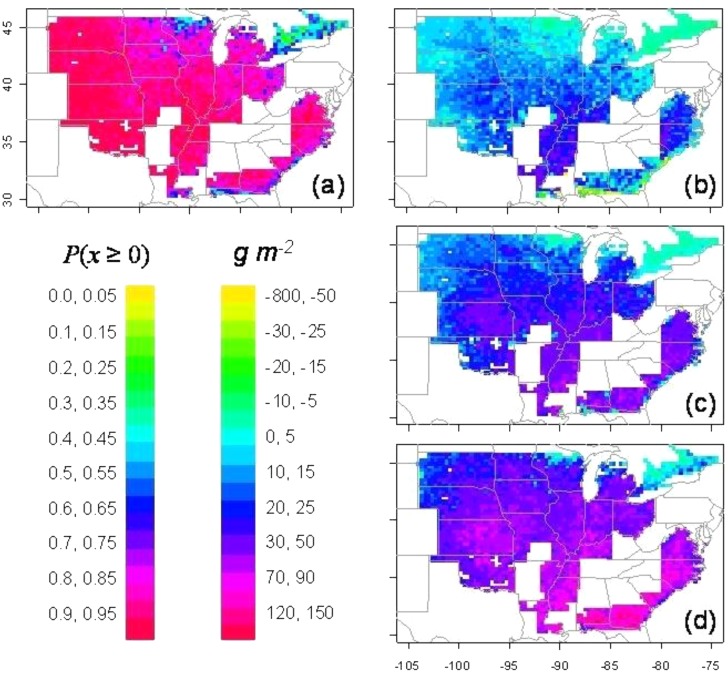
Simulation of yield change for soybean in the United States as a result of the introduction of a limited-transpiration trait (threshold = 2 kPa). The probability of yield increase over the 50 simulated years is shown in panel **(a)**. The actual yield increase at each location for percentile rankings of **(b)** 75% (wet), **(c)** 50% (median), and **(d)** 25% (dry) (Sinclair et al., 2010).

Simulations were also done on the impact of the limited-transpiration trait on soybean in the United States (Sinclair et al., 2010). The limited-transpiration response was invoked whenever VPD during the daily cycle was greater than 2 kPa. Simulations were done at each grid location (30 km × 30 km) over the United States based on 50 years of weather data. Due to the sensitivity of N_2_ fixation to soil drying, water conservation as a result of the limited-transpiration trait resulted in a high probability of yield increase of 85 % or greater for most locations (**Figure 2a**). Yields when ranked at each location showed yield increases at the 75 (wet), 50, and 25 (dry) percentile ranking in nearly all locations in the major areas of soybean production (**Figures 2b–d**). In the 25 percentile ranking, the yield increase ranged from 0.25 to 0.75 t ha^−1^. Similar simulations for soybean in Africa were done with the threshold for limited-transpiration trait at 1.8 kPa (Sinclair et al., 2014). Roughly half of the area in both East and West Africa had an 85% or greater probability of yield increase. The probability of a 70% or greater yield increase included all but the wettest and driest locations in Africa.

Geospatial assessments have also been done for several other crop species and locations. In South Asia, lentil (*Lens culinaris* Medik) was simulated with limited-transpiration thresholds of 2.2 and 1.1 kPa (Guiguitant et al., 2017). Limited-transpiration with a 1.1 kPa threshold had yield increase probability greater than 55% for much of the central zone of the South Asia region. Outside the central region, however, the simulations indicated the trait would not give consistent yield increase. Also, in South Asia Kholova et al. (2014) simulated the yield response of sorghum to modification in several plant traits. The limited-transpiration trait was found to have highest positive effect on crop yield of the studied traits. Over a wide range of yield levels, yields were increased up to 0.2 t ha^−1^.

The potential impact of the limited-transpiration trait on maize (*Zea mays* L.) yields in the United States was simulated by Messina et al. (2015). Using a 2.0 kPa limited-transpiration threshold, yield was generally increased in environments where yield without the trait was less than 10.5 t ha^−1^, with the greatest yield increases occurring at yield levels less than 6.5 t ha^−1^. Hence the greatest benefit in yield increase of the limited-transpiration trait was in the western regions of maize production in the United States, generally west of 95° west longitude. The results of these simulations are being used by Pioneer to guide the marketing of their AQUAmax hybrids, which have been shown to express the limited-transpiration trait.

### Assessment of Soil-Drying Sensitivity on Crop Yield

**FIGURE 3:**
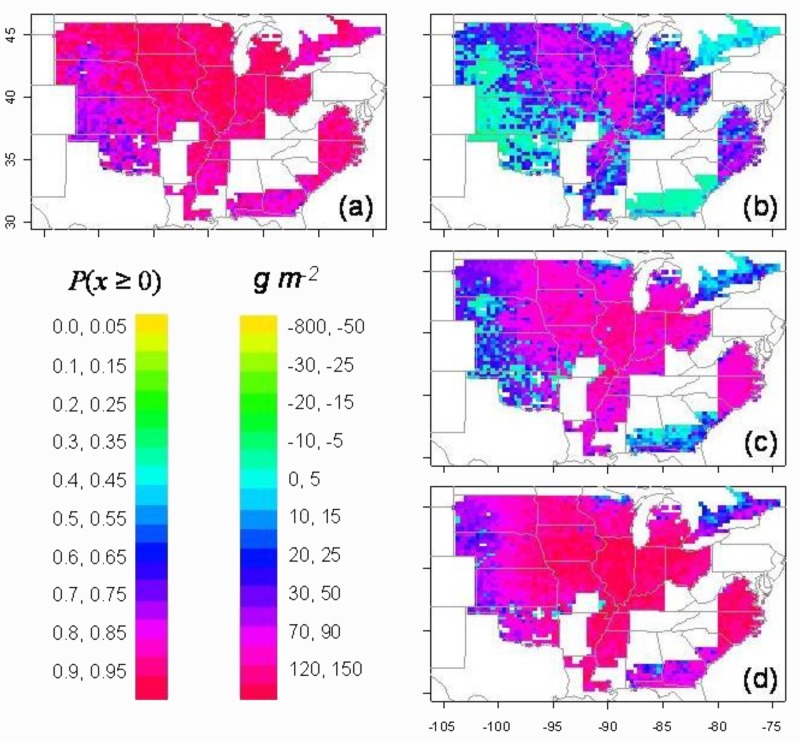
Simulation of yield change for soybean in the United States as a result of the introduction of early partial stomatal closure with soil drying (breakpoint at FTSW = 0.33). The probability of yield increase over the 50 simulated years is shown in panel **(a)**. The actual yield increase at each location for percentile rankings of **(b)** 75% (wet), **(c)** 50% (median), and **(d)** 25% (dry) (Sinclair et al., 2010).

Partial stomatal closure at a higher FTSW was simulated for maize grown at Columbia, MO. Yield in only 3 out of 20 simulated seasons was benefitted by initiating partial stomatal closure at higher soil water content than normally observed (Sinclair and Muchow, 2001). A much more extensive simulation of the response of soybean to higher FTSW for stomatal closure was included in the study for the United States described above by Sinclair et al. (2010). Due to the sensitivity to soil water deficit of symbiotic nitrogen fixation in soybean, the probability of yield increase was greater than 79% for three–fourths of the locations (**Figure 3a**). Yields when ranked at each location showed yield increases at the 75 (wet), 50, and 25 (dry) percentile rankings in nearly all locations in the major areas of soybean production (**Figures 3b–d**). Yields were increased especially in the drier growing seasons represented by the 25 percentile ranking. Therefore, available water was much more effectively used through the growing season as a result of the early initiation of stomatal closure at high FTSW.

## Effective Water Use

The above discussion of water conservations traits either by limited-transpiration rate under elevated VPD or by early decline in transpiration rate with soil drying showed the importance of shifting water use from earlier in the cropping season to later in the season, especially to the seed-fill period. Increasing availability of water to the crop at the end of the season enhances the possibility of sustained crop physiological activity, and hence, decreasing the impact of water deficit on reproductive growth. That is, the key response variable to increase yield as expressed in the phenomenological perspective of Eq. [1] is an increased HI.

There are, of course, additional approaches to water-conservation by crops other than those discussed above to achieve effective water use through a cropping season. One simple management approach that was empirically developed from field trails is to shift the cropping season to cooler periods when the VPD is lower and the overall water requirement is less. Another management approach is to select shorter-season cultivars so the cropping season can be completed before water deficits develop. In the Midsouth of the United States, a major shift in soybean management to increase yields by avoiding drought, which commonly develops at the end of July (Purcell et al., 2003), was achieved by using a combination of early sowing and early-maturing cultivars (Bowers, 1995; Heatherly, 1999).

In contrast to the soybean experience, simulations of lentil production in east Africa indicated longer-season cultivars had a higher probability of yield increase (Ghanem et al., 2015). The longer-season cultivars were better suited to take full advantage of all rainfall to attain more effective water use. The optimum sowing date varied substantially across the region so that full consideration of the rainfall pattern is required to determine the appropriate crop sowing date for each location.

Other plant traits can potentially be altered to decrease plant water use in the early part of the cropping season. Lower leaf photosynthesis rates early in the growing season as a result of low leaf nitrogen content and low stomatal conductance, for example, will result in water conservation for late season water use. Slower leaf area development will also decrease the light intercepting leaf area index so early-season transpiration rate is lessened. These traits are associated with the relative performance of maize, sorghum and millet with increasingly dry conditions favoring sorghum and then millet (Sinclair and Weiss, 2010). Slow root extension in the soil will also limit early season transpiration rate in favor of later season water use. The simulations of soybean production across the United States showed such traits would increase the probability of yield increase for much of the soybean production area (Sinclair et al., 2010). Of course, an important consideration in these early-season water conservation traits is minimization of water loss due to soil evaporation and competing weeds.

## Perspective

The phenomenological equation indicating the importance of TE turns out to be very complex at the mechanistic level. As found in the derivation of Tanner and Sinclair (1983), TE in the phenomenological equation is actually dependent on several physical and physiological variables as well as the environment. As a minimum, accounting for atmospheric humidity conditions is essential as was done in deWit’s analysis 60 years ago. As shown in deWit’s results, little variation within a crop species in TE normalized for VPD seemed to exist.

Even “intrinsic” TE may offer minimum insight. This term is usually assumed to be static, i.e., constant over a range a conditions. In fact, environmental variation over the growing season is likely to introduce instability that challenges the data requirements to fully establish intrinsic TE. Simply obtaining sufficient observations of intrinsic TE in a breeding population may be a major problem. The carbon isotope discrimination technique was developed in an effort to overcome this problem but the approach in practice is essentially empirical and is vulnerable to poor relationships with TE (Sinclair, 2012; Vadez et al., 2014).

For crop improvement, a major limitation in using “intrinsic” TE is that the potential improvement may be quite limited for crop species that already have been subjected to selection focused simply on improved yield in water-deficit environments. Intrinsic TE is limited by C_i_, which appears to have well-defined limits with maximum values of C_i_/C_a_ of approximately 0.3 and 0.7 for C4 and C3 species, respectively. Of course, if current cultivars deviate substantially above these limits, then potential for TE improvement clearly exist. Even in this case, the more direct breeding approach may be simply selection for superior yield under specified, water-limited conditions.

Rather than focus on TE, much greater return in yield improvement is expected from consideration of the temporal dynamics of water use through the growing season to improve the effective use of available water (Blum, 2009; Sinclair, 2012). This approach to a large extent likely focuses on decreasing water use early in the season or drying cycles to increase water availability to sustain physiological activity through seed fill. Drought during seed fill, especially those water deficits causing early termination of seed growth, can have large negative impacts on yield. As discussed above, simulation studies have shown under a range of conditions that improving plant water-conservation traits can results in high probabilities of yield increase, and absolute yield increases in drier environments can be substantial. Research to introduce these various options into commercial cultivars for increasing effective water use seems much more promising than the century-old, generally unfulfilled quest to improve static TE.

## Author Contributions

The author confirms being the sole contributor of this work and approved it for publication.

## Conflict of Interest Statement

The author declares that the research was conducted in the absence of any commercial or financial relationships that could be construed as a potential conflict of interest.

## References

[B1] Abbate P. E. Dardanelli J. L. Cantarero M. G. Maturano M. Melchiori R. J. M. Suero E. E. (2004). Climate and water availability effects on water-use efficiency in wheat. *Crop Sci.* 44 474–483. 10.1111/plb.12883 30059605

[B2] Blum A. (2009). Effective use of water (EUW) and not water-use efficiency (WUE) is the target of crop yield improvement under drought stress. *Field Crops Res.* 112 119–123. 10.1016/j.fcr.2009.03.009

[B3] Bowers G. R. (1995). An early season production system for drought avoidance. *J. Prod. Agric.* 8 112–119. 10.2134/jpa1995.fpage

[B4] Bunce J. A. (2005). What is the usual internal carbon dioxide concentration in C4 species under midday field conditions? *Photosynthetica* 43 603–608. 10.1007/s11099-005-0094-y

[B5] Devi M. J. Sinclair T. R. Vadez V. (2010). Genotypic variation in peanut for transpiration response to vapor pressure deficit. *Crop Sci.* 50 191–196. 10.2135/cropsci2009.04.0220

[B6] Devi M. J. Sinclair T. R. Vadez V. Krishnamurthy L. (2009). Peanut genotypic variation in transpiration efficiency and decreased transpiration during progressive soil drying. *Field Crops Res.* 114 280–285. 10.1016/j.fcr.2009.08.012

[B7] deWit C. T. (1958). *Transpiration and crop yield.* Verslag Landbouwk Onderz 64.6 Wageningen: Institute for Biological & Chemical Research.

[B8] Ghanem M. E. Marrou H. Birada C. Sinclair T. R. (2015). Production potential of lentil (*Lens culinaris* Medik.) in East Africa. *Agric. Syst.* 137 24–38. 10.1016/j.agsy.2015.03.005

[B9] Guiguitant J. Marrou H. Vadez V. Gupta P. Kumar S. Soltani A. (2017). Relevance of limited-transpiration trait for lentil (*Lens culinaris* Medik.) in South Asia. *Field Crops Res.* 209 96–107. 10.1016/j.fcr.2017.04.013

[B10] Heatherly L. G. (1999). “Early soybean production system (ESPS),” in *Soybean Production in the Midsouth*, eds Heatherly L. G. Hodges H. F. (Boca Raton, FL: CRS Press), 103–118.

[B11] Jackson P. Basnayake J. Inman-Bamber G. Lakshamanan P. Natarajan S. Stokes C. (2016). Genetic variation in transpiration efficiency and relationships between whole plant and leaf gas exchange measurements in *Saccharum* spp. and related germplasm. *J. Exp. Bot.* 67 861–871. 10.1093/jxb/erv505 26628517 PMC4737081

[B12] Kholova J. Murugesan T. Kaliamoorthy S. Malayee S. Baddam R. Hammer G. L. (2014). Modeling the effect of plant water use traits on yield and stay-green expression in sorghum. *Funct. Plant Biol.* 41 1019–1034. 10.1071/FP13355 32481055

[B13] Messina C. D. Sinclair T. R. Hammer G. L. Curan D. Thompson J. Oler Z. (2015). Limited-transpiration trait may increase maize drought tolerance in the US Corn Belt. *Agron. J.* 107 1978–1986. 10.2134/agronj15.0016

[B14] Passioura J. B. (1977). Grain yield, harvest index and water use of wheat. *J. Aus. Inst. Agric. Sci.* 48 117–121.

[B15] Purcell L. C. Sinclair T. R. McNew R. W. (2003). Drought avoidance assessment for summer annula crops using long-term weather data. *Agron. J.* 95 1566–1576. 10.2134/agronj2003.1566

[B16] Rebetzke G. J. Condon A. G. Richards R. A. Farquhar G. D. (2002). Selection for reduced carbon isotope discrimination increases aerial biomass and grain yield of rainfed bread wheat. *Crop Sci.* 42 739–745. 10.2135/cropsci2002.7390

[B17] Richards R. A. Passioura J. B. (1989). A breeding program to reduce the diameter of the major xylem vessel in the seminal roots of wheat and its effect on grain yield in rain-fed environments. *Aust. J. Agric. Res.* 40 943–950. 10.1071/AR9890943

[B18] Sadok W. Sinclair T. R. (2010). Genetic variability of transpiration response of soybean (*Glycine max* (L.) Merr.) shoots to leaf hydraulic conductance inhibitor AgNO3. *Crop Sci.* 50 1423–1430. 10.2135/cropsci2009.10.0575

[B19] Sinclair T. R. (2005). Theoretical analysis of soil and plant traits influencing daily plant water flux on drying soils. *Agron. J.* 97 1148–1152. 10.2134/agronj2004.0286

[B20] Sinclair T. R. (2012). Is transpiration efficiency a viable plant trait in breeding for crop improvement? *Funct. Plant Biol.* 39 359–365. 10.1071/FP11198 32480788

[B21] Sinclair T. R. (2017a). “Early partial stomata closure with soil drying,” in *Water-Conservations Traits to Increase Crop Yields in Water-Deficit Environments: Case Studies*, ed. Sinclair T. R. (Switzerland: Springer). 10.1007/978-3-319-56321-3

[B22] Sinclair T. R. (2017b). “Limited transpiration rate under elevated atmospheric vapor pressure deficit,” in *Water-Conservations Traits to Increase Crop Yields in Water-Deficit Environments: Case Studies*, ed. Sinclair T. R. (Switzerland: Springer). 10.1007/978-3-319-56321-3

[B23] Sinclair T. R. Hammer G. L. van Oosterom E. J. (2005). Potential yield and water-use efficiency benefits in sorghum from limited maximum transpiration rate. *Funct. Plant Biol.* 32 945–952. 10.1071/FP05047 32689190

[B24] Sinclair T. R. Marrou H. Soltani A. Vadez V. (2014). Soybean production potential in Africa. *Glob. Food Sec.* 3 31–40. 10.1016/j.gfs.2013.12.001

[B25] Sinclair T. R. Messina C. D. Beatty A. Samples M. (2010). Assessment across the United States of the benefits of altered soybean drought traits. *Agron. J.* 102 475–482. 10.2134/agronj2009.0195

[B26] Sinclair T. R. Muchow R. C. (2001). System analysis of plant traits to increase grain yield on limited water supplies. *Agron. J.* 93 263–270. 10.2134/agronj2001.932263x

[B27] Sinclair T. R. Weiss A. (2010). *Principles of Ecology in Plant Production*, 2 Edn. Wallingford: CAB International 10.1079/9781845936549.0000

[B28] Sinclair T. R. Zwieniecki M. A. Holbrook N. M. (2008). Low leaf hydraulic conductance associated with drought tolerance in soybean. *Physiol. Plant.* 132 446–451. 10.1111/j.1399-3054.2007.01028.x 18333998

[B29] Soltani A. Sinclair T. R. (2012). *Modeling Physiology of Crop Development, Growth and Yield*. Wallingford: CAB Intl. 10.1079/9781845939700.0000

[B30] Stiller W. N. Read J. J. Constable G. A. Reid P. E. (2005). Selection for water use efficiency traits in cotton breeding program: cultivar differences. *Crop Sci.* 45 1107–1113. 10.2135/cropsci2004.0545

[B31] Tanner C. B. Sinclair T. R. (1983). “Efficient water use in crop production: research or re-search?,” in *Limitation to Efficient Water Use in Crop Production*, eds Taylor H. M. Jordan W. R. Sinclair T. R. (Madison, WI: American Society of Agronomy), 1–27.

[B32] Tomeo N. J. Rosenthal D. M. (2017). Variable mesophyll conductance among soybean cultivars sets a tradeoff between photosynthesis and water-use efficiency. *Plant Phyiol.* 174 241–252. 10.1104/pp.16.01940 28270627 PMC5411144

[B33] Vadez V. Kholova J. Medina S. Kakkera A. Anderberg H. (2014). Transpiration efficiency: new insights into an old story. *J. Exp. Bot.* 65 6141–6153. 10.1093/jxb/eru040 24600020

